# Supplementary material for: Entanglement of orbital angular momentum in non-sequential double ionization
Source: Nat Commun. 2022 Aug 10;13:4706. doi: 10.1038/s41467-022-32128-z (PMC9365801; doi:10.1038/s41467-022-32128-z)
Supplement: Supplementary file 1 — Supplementary Information [file 41467_2022_32128_MOESM1_ESM.pdf]

Supplementary Information: Entanglement of Orbital Angular Momentum in Non-Sequential Double Ionization

Andrew S. Maxwell [1, 2, *] Lars Bojer Madsen [2] and Maciej Lewenstein [1, 3]

[1] ICFO-Institut de Ciencies Fotoniques, The Barcelona Institute of Science and Technology, Av. Carl Friedrich Gauss 3, 08860 Castelldefels (Barcelona), Spain
[2] Department of Physics and Astronomy, Aarhus University, DK-8000 Aarhus C, Denmark
[3] ICREA, Pg. Lluís Companys 23, 08010 Barcelona, Spain

We discuss the following topics: (i) An in-depth introduction to the logarithmic negativity and comparison to other entanglement measure. (ii) The incoherent averaging of the laser focal volume and its effect on the overall entanglement is considered. (iii) The basic expressions and estimates of entanglement for the electron-impact mechanism for non-sequential double ionization.

SUPPLEMENTARY NOTE 1: LOGARITHMIC NEGATIVITY VS OTHER ENTANGLEMENT MEASURES

Many commonly used entanglement measures, like the purity and entropy of entanglement (and other Renyi entanglement entropies) [1], use the property that the reduced density matrix of an entangled pure state is a mixed state. However, this means they are not directly applicable to an entangled mixed state system without non-trivial extensions. This involves the convex roof extension [2], which extends pure state measures to mixed states. An extension of entropy of entanglement to mixed states is the entropy of formation, which is defined using the concurrence [3]. However, computing the concurrence for mixed state systems of a higher dimensional Hilbert space than two qubits is not simple.

The logarithmic negativity [4], on the other hand, uses the properties of the positive partial transpose (PPT) criterion [5, 6]. The PPT criterion applies the partial transpose to the system and tests for entanglement by checking if any of the eigenvalues become negative, which would suggest the density matrix after partial transposition no longer corresponds to any 'real' quantum state. The partial transpose, is physically equivalent to the time reversal of one of the subsystems [7], i.e., one of the electrons. For two separable particles, the partial time reversal or transpose of a subsystem still corresponds to a 'real' quantum state, as either of the quantum states for the particles is time-reversible, hence the eigenvalues remain positive. However, for an entangled system, performing a partial transpose/time reversal on a single subsystem is not guaranteed to map back to a 'real' quantum state and will often result in negative eigenvalues. This is PPT entanglement, one of the most generally applicable entanglement criteria. The negativity and logarithmic negativity generalize this into entanglement measures, which employ the sum of the absolute value of the negative eigenvalues. Such an entanglement measure is very simple to compute and directly applicable to mixed states. Given that the PPT condition is necessary but not sufficient for separability, there are entangled states that will not lead to negative eigenvalues. Hence, these states have zero logarithmic negativity.

The entropy of entanglement is computed via the von Neumann entropy on the reduced density matrix, tracing over one of the subsystems. Thus, it has clear interpretation in terms of entropy and information theory, which makes it a popular choice for quantifying entanglement. On the other hand, the logarithmic negativity gives an upper bound to distillable entanglement [4]. Distillable entanglement quantifies how many copies of a state would be required to produce a maximally entangled state, which in the case of a pure state equates to the entropy of entanglement. Another way to understand logarithmic entanglement is that is quantifies the entanglement cost of creating a state by positive-partial-transpose-preserving operations. These are operations that map a state with positive partial transpose onto another state with a positive partial transpose. [8]. Direction comparison of the logarithmic negativity with other entanglement measures is given in Ref. [9].

In conclusion, we consider the logarithmic negativity as it is one of the simplest and powerful entanglement measures to compute, which is valid for mixed state systems. While, other commonly employed entanglement measures such as the entropy of entanglement are not directly applicable to mixed states.

SUPPLEMENTARY NOTE 2: FOCAL AVERAGING

In this section we consider the effect of incoherent averaging on the momentum distribution, density matrix and entanglement, in the form of focal averaging. The focal averaging used for the momentum-dependent probability distributions has been discussed extensively in Refs. [10, 11]. The extension to density matrices takes an almost identical form. The basic equations for focal averaging are given by integrating the ionization rate for a specific intensity over the focal volume and duration of the laser field to get a measure proportional to the num-

* andrew.maxwell@phys.au.dk

ber of electrons at a specific momentum in experiment

$$N(p_\parallel, p'_\parallel) \propto \int dt \int d^3\mathbf{r}\Omega(p_\parallel, p'_\parallel, I(r_{||}, r_\perp, t)), \quad (1)$$

where $\Omega(p_\parallel, p'_\parallel, I)$ is the probability $|M(p_\parallel, p'_\parallel)|^2$ given at a specific intensity I and $I(r_{||}, r_\perp, t)$ gives an approximation to the laser beam intensity profile

$$I(r_{||}, r_\perp, t) = I_0 \frac{d_0}{d(r_{||})} \exp\left(-\frac{2r_\perp^2}{d(r_{||})^2}\right) \exp\left(\frac{(t - r_{||}/c)^2}{\tau^2}\right) \quad (2)$$

with $d(z) = d_0 \left[1 + (z/z_0)^2\right]^{\frac{1}{2}}$, where $d_0 = \sqrt{\lambda z_0/\pi}$ is the beam waist, I_0 is the peak intensity, z_0 is the Rayleigh length, λ is the wavelength, and c is the speed of light. Then Eq. (1) may be simplified and parameterized in terms of an integral over the laser intensity

$$N(p_\parallel, p'_\parallel) \propto \int_0^{I_0} dI f(I) \Omega(p_\parallel, p'_\parallel, I), \quad (3)$$

where

$$f(I) = \frac{1}{I} \int_0^{\eta} d\eta (1 + \eta^2) \ln\left(\frac{I_0}{I(1+\eta^2)}\right)^{1/2}. \quad (4)$$

In order to take into account the focal volume for density matrices, a similar process must be performed. We can write the density matrix corresponding to a specific laser intensity as $\rho(I)$, now the mixed state that accounts for all intensities over the focal volume is the weighted sum over all intensities in the volume, where the weights are given by $f(I)$ such that

$$\rho_{\text{focal}} \propto \int_0^{I_0} dI f(I) \rho(I). \quad (5)$$

In Fig. 3 in the main manuscript, we show results without focal averaging, here in Supplementary Fig. 1, we show the corresponding focally averaged momentum distributions as well as density matrices. As expected, the interference fringes are reduced in the momentum distributions. As an example, the ratio between the central peak and its adjacent trough in magnesium halves from 6, in Fig. 3(a) in the main manuscript, to 3 in Supplementary Fig. 1(a). But what effect does this have on the entanglement? Interestingly, the logarithmic negativity, labelled on the density matrix in Supplementary Fig. 1(b) and Supplementary Fig. 1(d), stays relatively high. In fact, for magnesium, it slightly increases. The reason for this is because, in the case of magnesium, the SFA predicts that at lower intensities the logarithmic negativity increases to $E_{\mathcal{N}} = 1.06$ quite rapidly as lower energy excitation channels ($3p$ and $3d$) begin to play a role. We did not focus on these lower intensities due to their higher Keldysh parameter, outside our search region. In Supplementary Fig. 1(b), the elements $|0, 0\rangle$ and $|\pm1, \mp1\rangle$ increase in magnitude and there is a change in the phase associated with more contribution from these additional states. In contrast, for beryllium, in Supplementary Fig. 1(d), we see a small reduction in the same element and the phase remains the same. In general, the elements associated with $|\pm1, \mp1\rangle$ and $|\mp1, \pm1\rangle$ remain coherent, and the entanglement is robust to incoherent averaging over intensity.

Interference in NSDI and the RESI mechanism has been a significant issue, see e.g., [11–13]. Thus, it is important to note that the fine fringes in Supplementary Fig. 1 of this supplement do remain visible even after focal averaging. From experimental results, such as [14], which has been widely used as a benchmark reference by theory, low statics make it unclear if such fringes survive or not. It is important to note that the fine fringes, visible in Supplementary Fig. 1, are not due to the coherence between channels of excitation, but to coherence between wavepackets which ionize at different times in the pulse. So these fringes are not related to the entanglement studied here. Additionally, the inclusion of rescattering in the second electron, see Ref. [15], could actually lead to further washing out of these fringes.

SUPPLEMENTARY NOTE 3: ELECTRON-IMPACT IONIZATION

In this section, we give some details on the entanglement properties in electron-impact ionization (EI) in comparison to the recollision with subsequent ionization (RESI) mechanism. Here, we will formulate the strong field approximation (SFA) transition amplitude for EI ionization, see [16] for more details, many features are similar to the derivation of the RESI transition amplitude in the Methods section of the main manuscript. As before, the transition amplitude is symmetrized to account for the electron indistinguishability given an initial spin singlet state

$$M^{\text{EI}}(\tilde{\mathbf{p}}, \tilde{\mathbf{p}}') = \frac{1}{\sqrt{2}} \left(M^{\text{EI}}_{\text{unsym}}(\tilde{\mathbf{p}}, \tilde{\mathbf{p}}') + M^{\text{EI}}_{\text{unsym}}(\tilde{\mathbf{p}}', \tilde{\mathbf{p}})\right). \quad (6)$$

For EI the unsymmetrized transition amplitude is given by

$$M^{\text{EI}}_{\text{unsym}}(\tilde{\mathbf{p}}, \tilde{\mathbf{p}}') = \int d^2t \int d^3\tilde{\mathbf{k}} V_{\tilde{\mathbf{p}}\tilde{\mathbf{p}}',\tilde{\mathbf{k}}0} V_{\tilde{\mathbf{k}}0,0} \exp[iS(\mathbf{p}, \mathbf{p}', \mathbf{k}, t, t')], \quad (7)$$

where

$$\int d^2t \equiv \int_{-\infty}^{\infty} dt \int_{-\infty}^{t} dt' \quad (8)$$

and

$$S(\mathbf{p}, \mathbf{p}', \mathbf{k}, t, t') = I_p^{10} t' + I_p^{20} t - \int_{t'}^{t} \frac{[\mathbf{k} + \mathbf{A}(\tau)]^2}{2} d\tau - \int_t^{\infty} \frac{[\mathbf{p} + \mathbf{A}(\tau)]^2}{2} d\tau - \int_t^{\infty} \frac{[\mathbf{p}' + \mathbf{A}(\tau)]^2}{2} d\tau. \quad (9)$$

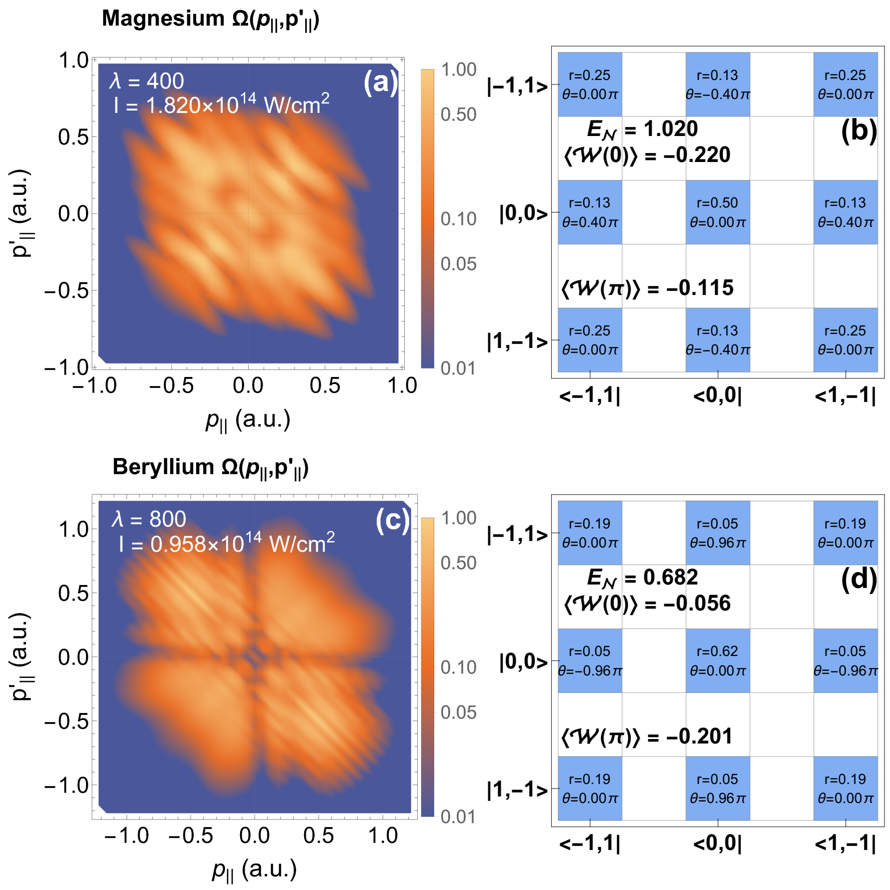

Supplementary Fig. 1. **Focally averaged momentum distributions and density matrices**. Same as Fig. 3 in the main manuscript, but now the focal volume of the laser is accounted for. Focal averaged distributions, panels (a) and (c), and focal averaged density matrices, panels (b) and (d). The logarithmic negativity and entanglement witness values are recalculated and printed on the top right of panels (b) and (d).

Here, I_p^{10} and I_p^{20} are the one-electron ionization potentials corresponding to removing an electron from the two-electron ground state $|0\rangle$ and from the state where one electron is bound and the other is the continuum $|\mathbf{p},0\rangle$, respectively. For more details on the notation see the Methods section of the main manuscript. The prefactors, given by

$$\begin{aligned} V_{\tilde{\mathbf{k}}0,0} &= \langle \tilde{\mathbf{k}}(t''),0|V|0\rangle \\ &= \frac{1}{(2\pi)^{3/2}}\int d^3\tilde{\mathbf{r}}\; V(\tilde{\mathbf{r}})e^{-i\tilde{\mathbf{k}}(t'')\cdot\tilde{\mathbf{r}}}\psi_{10}(\tilde{\mathbf{r}}), \end{aligned} \quad (10)$$

$$\begin{aligned} V_{\tilde{\mathbf{p}}\tilde{\mathbf{p}}',\tilde{\mathbf{k}}0} &= \langle \tilde{\mathbf{p}}(t'),\tilde{\mathbf{p}}'(t')|V_{12}|\tilde{\mathbf{k}}(t'),0\rangle \\ &= \frac{1}{(2\pi)^{5/2}}\iint d^3\tilde{\mathbf{r}}'d^3\tilde{\mathbf{r}}\exp[-i(\tilde{\mathbf{p}}-\tilde{\mathbf{k}})\cdot\tilde{\mathbf{r}}] \\ &\quad\times\exp(-i\tilde{\mathbf{p}}'\cdot\tilde{\mathbf{r}}')V_{12}(\tilde{\mathbf{r}},\tilde{\mathbf{r}}')\psi_{20}(\tilde{\mathbf{r}}'), \end{aligned} \quad (11)$$

describe the ionization and recollision steps, respectively. Where V is the singly charged binding potential and V_{12} is the electron-electron interaction. Here, $\tilde{\mathbf{p}}(t)$, $\tilde{\mathbf{p}}'(t)$ and $\tilde{\mathbf{k}}(t)$ are defined according to $\tilde{\mathbf{k}}(t)=\tilde{\mathbf{k}}+\tilde{\mathbf{A}}(t)$ or $\tilde{\mathbf{k}}(t)=\tilde{\mathbf{k}}$ in the length or velocity gauge, respectively. Throughout this work, we have employed the velocity gauge.

The same overall OAM conservation must be obeyed, as in the RESI case, $m+m'=l_e+l_e'$. This identity and further section rules are encoded in the Fourier series decomposition of the prefactors.

$$V_{\tilde{\mathbf{k}}0,0} = e^{im\phi_{\mathbf{k}}}\tilde{V}_{\mathbf{k}0,0} \quad (12)$$

$$V_{\tilde{\mathbf{p}}\tilde{\mathbf{p}}',\tilde{\mathbf{k}}0} = \sum_{\ell_p=-\infty}^{\infty}\sum_{m_p=-\ell_p}^{\ell_p} e^{i(m'-m_p)\phi}e^{im_p\phi'}\tilde{V}^{\ell_p}_{\mathbf{p}\mathbf{p}',\mathbf{k}0}. \quad (13)$$

Now, using Eq. (6) in the main manuscript we may write the OAM SFA transition amplitude

$$M^{EI}_{l_e,l_e'}(\mathbf{p},\mathbf{p}') = i^{-(l_e+l_e')}\delta_{m,0}\delta_{m',l_e'+l_e}\tilde{M}(\mathbf{p},\mathbf{p}') \quad (14)$$

with

$$\tilde{M}(\mathbf{p},\mathbf{p}') = \sum_{\ell_p=-\infty}^{\infty}\int d^2t\int d^2\mathbf{k}\tilde{V}^{\ell_p}_{\mathbf{p}\mathbf{p}',\mathbf{k}0}\tilde{V}_{\mathbf{k}0,0}e^{iS(\mathbf{p},\mathbf{p}',\mathbf{k},t,t')}. \quad (15)$$

Unlike the RESI case, the final OAM values are not completely determined by selection rules from the prefactors. From Eq. (14) we can determine the selection rules $m = 0$ and $m' = l_e + l_e'$, where l_e and l_e' may take any integer value satisfying this relation. First, we consider the cases of beryllium and magnesium, where $m' = 0$. From Eqs. (12–13) it is clear it is $\tilde{V}^{\ell_p}_{\mathbf{pp}',\mathbf{k}0}$ that determines the balance of final OAM states. Thus, in order to approximately quantify the entanglement, we may compute $\tilde{V}^{\ell_p}_{\mathbf{pp}',\mathbf{k}0}$ to estimate the ratio between the states $|0,0\rangle$ and $|\pm1,\mp1\rangle$, without resorting to computing the full transition amplitude. Specifically, given a state of the final form

$$|\psi\rangle = \alpha\,|0,0\rangle + \beta(|{-1},1\rangle + |1,-1\rangle), \tag{16}$$

using $\tilde{V}^{\ell_p}_{\mathbf{pp}',\mathbf{k}0}$, we may estimate α and β and use this to compute the logarithmic negativity. We pick momentum and field values that maximize the logarithmic negativity, and thus provide an approximate upper bound to the entanglement. We have done this and found the coefficient $|\alpha|$ is 2–3 orders of magnitude larger than $|\beta|$, which would correspond to logarithmic negativity of $E_{\mathcal{N}} < 0.06$ for beryllium and magnesium, far lower than the cases considered in the main body of the manuscript. Using a similar logic, we find for helium $E_{\mathcal{N}} < 0.2$.

For the cases of argon and neon, computing the same sum over $\tilde{V}^{\ell_p}_{\mathbf{pp}',\mathbf{k}0}$ for $m' = 0$, leads to the coefficient $|\alpha|$ to be 1–2 orders of magnitude larger than $|\beta|$. We estimate the logarithmic negativity to be $E_{\mathcal{N}} < 0.5$ for neon and $E_{\mathcal{N}} < 0.3$ for argon. However, the quantum magnetic number of the 'second' electron may take the values $m' = \pm1$, which lead to alternative final OAM states. These correspond to different ionic final states, and thus, will incoherently contribute to the process. To account for these processes, the density matrix for each of these cases must be summed, i.e., $\rho = \rho_{m'=-1} + \rho_{m'=0} + \rho_{m'=0}$. We have done this, but the $m' = \pm1$ channel have low probably and so this only marginally reduces the logarithmic negativity.

The calculations were computed at specific final momenta and do not account for tracing over momentum, so we expect the overall value of the logarithmic negativity to significantly reduce. Given the values for neon are not vanishingly small, it may warrant further study. However, it does not appear to be as fruitful as the RESI case, where at specific momenta pairs of electrons may be produced that maximize the entanglement, and excited state superposition protects against decoherence with the ion.

SUPPLEMENTARY REFERENCES

[1] R. Horodecki, P. Horodecki, M. Horodecki, and K. Horodecki, Quantum entanglement, Rev. Mod. Phys. **81**, 865 (2009).

[2] R. F. Werner, Quantum states with Einstein-Podolsky-Rosen correlations admitting a hidden-variable model, Phys. Rev. A **40**, 4277 (1989).

[3] S. A. Hill and W. K. Wootters, Entanglement of a Pair of Quantum Bits, Phys. Rev. Lett. **78**, 5022 (1997).

[4] G. Vidal and R. F. Werner, Computable measure of entanglement, Phys. Rev. A **65**, 032314 (2002).

[5] A. Peres, Separability Criterion for Density Matrices, Phys. Rev. Lett. **77**, 1413 (1996).

[6] P. Horodecki, Separability criterion and inseparable mixed states with positive partial transposition, Physics Letters A **232**, 333 (1997).

[7] H. Shapourian, K. Shiozaki, and S. Ryu, Partial time-reversal transformation and entanglement negativity in fermionic systems, Phys. Rev. B **95**, 165101 (2017).

[8] K. Audenaert, M. B. Plenio, and J. Eisert, Entanglement Cost under Positive-Partial-Transpose-Preserving Operations, Phys. Rev. Lett. **90**, 027901 (2003).

[9] A. Miranowicz, S. Ishizaka, B. Horst, and A. Grudka, Comparison of the relative entropy of entanglement and negativity, Phys. Rev. A **78**, 052308 (2008).

[10] R. Kopold, W. Becker, M. Kleber, and G. G. Paulus, Channel-closing effects in high-order above-threshold ionization and high-order harmonic generation, J. Phys. B: At. Mol. Opt. Phys. **35**, 217 (2002).

[11] A. S. Maxwell and C. Figueira de Morisson Faria, Controlling Below-Threshold Nonsequential Double Ionization via Quantum Interference, Phys. Rev. Lett. **116**, 143001 (2016).

[12] X. Hao, J. Chen, W. Li, B. Wang, X. Wang, and W. Becker, Quantum Effects in Double Ionization of Argon below the Threshold Intensity, Phys. Rev. Lett. **112**, 073002 (2014).

[13] W. Quan, X. Hao, Y. Wang, Y. Chen, S. Yu, S. Xu, Z. Xiao, R. Sun, X. Lai, S. Hu, M. Liu, Z. Shu, X. Wang, W. Li, W. Becker, X. Liu, and J. Chen, Quantum interference in laser-induced nonsequential double ionization, Phys. Rev. A **96**, 032511 (2017).

[14] M. Kübel, K. J. Betsch, N. G. Kling, A. S. Alnaser, J. Schmidt, U. Kleineberg, Y. Deng, I. Ben-Itzhak, G. G. Paulus, T. Pfeifer, J. Ullrich, R. Moshammer, M. F. Kling, and B. Bergues, Non-sequential double ionization of Ar: From the single- to the many-cycle regime, New Journal of Physics **16**, 033008 (2014).

[15] X. Hao, Y. Bai, C. Li, J. Zhang, W. Li, W. Yang, M. Liu, and J. Chen, Recollision of excited electron in below-threshold nonsequential double ionization, Commun Phys **5**, 31 (2022).

[16] C. Figueira de Morisson Faria, T. Shaaran, X. Liu, and W. Yang, Quantum interference in laser-induced nonsequential double ionization in diatomic molecules: Role of alignment and orbital symmetry, Phys. Rev. A **78**, 043407 (2008).
